# Nitroimidazole-based inhibitors DTP338 and DTP348 are safe for zebrafish embryos and efficiently inhibit the activity of human CA IX in *Xenopus* oocytes

**DOI:** 10.1080/14756366.2018.1482285

**Published:** 2018-06-17

**Authors:** Ashok Aspatwar, Holger M. Becker, Nanda Kumar Parvathaneni, Milka Hammaren, Aleksandra Svorjova, Harlan Barker, Claudiu T. Supuran, Ludwig Dubois, Philippe Lambin, Mataleena Parikka, Seppo Parkkila, Jean-Yves Winum

**Affiliations:** a Faculty of Medicine and Life Sciences, University of Tampere, Tampere, Finland;; b Department of Physiological Chemistry, University of Veterinary Medicine Hannover, Hannover, Germany;; c Department of Radiotherapy, The M-Lab Group, GROW – School for Oncology and Developmental Biology, Maastricht University Medical Centre, Maastricht, The Netherlands;; d Institut des Biomolécules Max Mousseron (IBMM) UMR 5247 CNRS, ENSCM, Université de Montpellier, MontpellierCedex 05, France;; e NEUROFARBA Department, Section of Pharmaceutical and Nutraceutical Sciences, University of Florence, Polo Scientifico, Firenze, Italy;; f Fimlab Ltd. and Tampere University Hospital, Tampere, Finland

**Keywords:** Nitroimidazoles, carbonic anhydrase IX, zebrafish, *Xenopus* oocytes, *in vivo* inhibition

## Abstract

Carbonic anhydrase (CA) IX is a hypoxia inducible enzyme that is highly expressed in solid tumours. Therefore, it has been considered as an anticancer target using specific chemical inhibitors. The nitroimidazoles DTP338 and DTP348 have been shown to inhibit CA IX in nanomolar range *in vitro* and reduce extracellular acidification in hypoxia, and impair tumour growth. We screened these compounds for toxicity using zebrafish embryos and measured their *in vivo* effects on human CA IX in *Xenopus* oocytes. In the toxicity screening, the LD_50_ for both compounds was 3.5 mM. Neither compound showed apparent toxicity below 300 µM concentration. Above this concentration, both compounds altered the movement of zebrafish larvae. The IC_50_ was 0.14 ± 0.02 µM for DTP338 and 19.26 ± 1.97 µM for DTP348, suggesting that these compounds efficiently inhibit CA IX *in vivo*. Our results suggest that these compounds can be developed as drugs for cancer therapy.

## Introduction

Carbonic anhydrase (CA) IX is a zinc-containing metalloenzyme that efficiently catalyses the reversible hydration of CO_2_ to H^+^ and HCO_3_^− 1–3^. CA IX is highly expressed in hypoxic tumours where it is involved in the regulation of acid-base balance and cell-to-cell adhesion^4–9^. In normal tissues, CA IX shows limited expression in the gastric mucosa, gall bladder, bile ducts and small intestine^8^^,^^10^. The high expression of CA IX in hypoxic tumours and the associated molecular events contribute to metastatic phenotype and resistance to anticancer drugs, promoting the survival of cancer cells as well as tumour progression^11^. CA IX is an important contributor to the tumour microenvironment where it participates in ion transport across the plasma membrane. The enzyme thus protects the tumour cells from intracellular acidosis by facilitating the export of acidic metabolic products^5^^,^^8^.

Hypoxic tumours do not respond well to general chemotherapy and radiotherapy in many cases^8^^,^^9^^,^^12^. Inhibition of CA IX is considered a promising adjunct therapeutic option as it has been shown to sensitize hypoxic tumours to existing therapies^13^. Like the other α-CAs, CA IX can be inhibited by anions, sulfonamides, sulfamides and sulfamates^14^. Recently, many inhibitors have been identified that inhibit CA IX in low nanomolar quantities^2^^,^^15^. Binding of some inhibitors to CAs has been investigated using X-ray crystallography, and it has been shown that they are membrane impermeable^16^^,^^17^.

**Figure 1.**
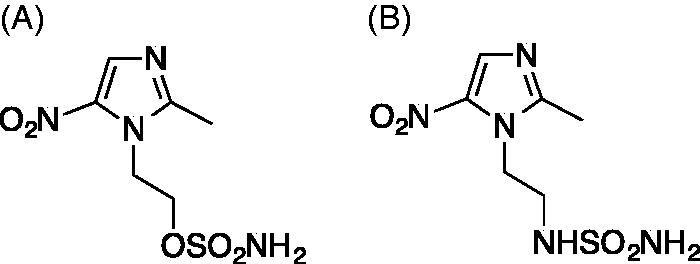
Chemical structures of the compounds used in the study. (A) The nitroimidazole DTP338 (compound 9 in the original study)^18^ inhibits human CA IX showed inhibition constant Ki 8.3 nM *in vitro*^18^. (B) The nitroimidazole DTP348 (compound 7 in original study) inhibitions human of CA IX *in vitro* at the concentration of 20.4 nM^18^.

In the pursuit of developing novel methods for the treatment of hypoxic tumours, we designed and synthesized a series of nitroimidazoles as radio/chemosensitizing agents, targeting the tumour-associated CA IX^18^. Among these compounds are two nitroimidazoles, DTP338 and DTP348 (Figure 1), that were synthesized by incorporating a sulfamate or sulfamide and moiety as zinc-binding groups (ZBGs) respectively, as described earlier by our group^19^^,^^20^. The *in vitro* inhibition kinetics showed that both of these compounds inhibit the activity of human CA IX in nanomolar quantities^18^. Further studies involving cancer cells showed that the inhibitors reduced hypoxia-induced extracellular acidosis in two different cell lines^18^. The lead compound DTP348 sensitized the CA IX containing tumours when treated in combination with doxorubicin^18^.

Before the newly synthesized compounds are taken into clinical trials and developed further as therapeutic agents, it is important to characterize them for safety and toxicity preclinically using cell and animal models. In the past, nitroimidazoles have shown significant clinical toxicity^21^^,^^22^. However, to date, none of the present agents have been screened for toxicity and safety using an animal model, and no *in vivo* inhibition profiles of nitroimidazoles against human CA IX have been demonstrated using an eukaryotic cell model.

In the recent past, zebrafish has emerged as a vertebrate animal model for determining drug-induced toxicity in a preclinical drug development process^23–26^. The zebrafish provides many advantages compared to the other vertebrate model organisms. These include high fecundity, rapid development *ex utero*, and the ease of maintaining large numbers in a small space. Administration of drug is easy as the embryos absorb the drug molecules from the water through their skin and gills^24^. Similarly, the *Xenopus laevis* has been widely used as an animal model in molecular and physiological research, especially with heterologously expressed proteins in the oocytes^27^. The oocytes have advantages, such as easy manipulation due to the large cell size (1.1–1.33 mm), convenient laboratory maintenance, and the oocytes can be obtained in large numbers^27^. The results of electrophysiological measurements obtained in *Xenopus* oocytes are not affected by the activity of endogenous proteins, making the oocytes versatile tools for biochemical applications. Similarly, native *Xenopus* oocytes contain undetectably low level of CA activity^28^. Therefore, the injections of cRNA coding for human CA isozyme into oocytes allow the expression of the target CA and investigation of CA inhibition in the live biological system. The inhibitory properties of ethoxzolamide (EZA), a membrane-permeable and nonselective sulfonamide CA inhibitor, against CA II, CA IV, and CA IX have been reported in oocytes^29–31^. Thus, *Xenopus* oocyte is a promising animal model to study the roles of CAs and characterize the affinity and selectivity of human CA inhibitors in the living eukaryotic cell with fully matured target CA isozyme.

In the present study, we used zebrafish as a vertebrate model organism and evaluated the safety and toxicity of the two nitroimidazole-based CA inhibitors DTP338 and DTP348, studied the inhibitory properties of these compounds *in vitro* using *Mycobacterium marinum* and studied their efficiency for inhibition of human CA IX *in vivo* in *Xenopus* oocytes. Figure 1 shows the structures of the inhibitors that were used in this study.

## Materials and methods

### Inhibitors

The two nitroimidazoles DTP338 and DTP348 (Figure 1(A,B)) used in the study were synthesized by some of our group and *in vitro* inhibition studies against several human CAs have been described previously^18^^,^^19^. Nitroimidazole DTP338, a sulfamate analogue was prepared from starting material, metronidazole, using a direct sulfamoylation procedure as described earlier^20^. The nitroimidazole sulfamide DTP348, and was obtained by a procedure reported earlier^18^^,^^19^. Both nitroimidazoles were investigated *in vitro* as specific inhibitors of human CA IX enzyme and found to inhibit the enzyme in sub-nanomolar range^18^. The compounds DTP338 and DTP348 were dissolved in dimethyl sulfoxide (DMSO) to prepare 100 mM stock solutions. Before exposing the 1 day post fertilization (dpf) embryos to the different dilutions of the compounds, a series of dilutions of each compound were prepared using embryonic medium [5.0 mM NaCl, 0.17 mM KCl, 0.33 mM CaCl_2_, 0.33 mM MgSO_4_, and 0.1% w/v Methylene Blue (Sigma-Aldrich, Germany)].

### Maintenance of zebrafish

Wild type zebrafish of the AB strain were maintained at 28.5 °C in an incubator, as described previously^25^. The 1–2 hour post fertilization (hpf) embryos were rinsed with embryonic medium and kept in the incubator at 28.5 °C overnight. The toxicity experiments were performed using the fish that were 24 hpf. All the zebrafish experiments were performed at the zebrafish core facility, University of Tampere, Finland.

### Ethical statement

The zebrafish core facility at the University of Tampere has an establishment authorization granted by the National Animal Experiment Board (ESAVI/7975/04.10.05/2016). All experiments using zebrafish were performed according to the Provincial Government of Eastern Finland, Social and Health Department of Tampere Regional Service Unit protocol # LSLH-2007–7254/Ym-23. Care was taken to ameliorate suffering by euthanizing the zebrafish by prolonged immersion in a petri dish containing an overdose of Tricaine (Sigma-Aldrich, St. Louis, MO, USA) before fixing in buffered formaldehyde for histochemical analysis.

### Toxicological studies of nitroimidazole compounds DTP338 and DTP348

#### Determination of LD_50_

For determining the LD_50_ values of the DTP338 and DTP348 compounds we used eight groups of zebrafish for each concentration of the compound, with sixty 1 dpf zebrafish per group. In each group, the larvae were exposed to different concentrations of the compound that ranged from 10 µM to 3.5 mM. The dose response curve (DRC) was calculated using DRM of the DRC R package^32^. As control groups, we used an equal number of wild type larvae not treated with any drug or compound and 1% DMSO treated larvae. The experiments were carried out in 24-well plates (Corning V R Costar V R cell culture plates). In each well, we placed ten 1 dpf embryos in 1 ml of embryonic medium containing either a diluted inhibitor, 1% DMSO diluted in embryonic medium or embryonic medium with no inhibitor. A minimum of three sets of experiments were carried out for each inhibitor. Survival of the embryos was checked every 24 h until five days after the first exposure.

#### Phenotypic analysis of control and inhibitor-treated larvae

To assess the effect of inhibitors on the embryos after five days of exposure to the inhibitors, we analysed seven observable phenotypic parameters: (1) movement pattern, (2) yolk sack utilization, (3) hatching, (4) heartbeat, (5) body shape, (6) swim bladder development and (7) oedema using a stereo microscope and recorded the observations for each group. The images of the developing larvae were taken using a Lumar V1.12 fluorescence microscope attached to a camera (Carl Zeiss MicroImaging GmbH, Göttingen, Germany). The images were analysed with AxioVision software versions 4.7 and 4.8.

#### Swim pattern analysis

The movement of the zebrafish larvae was tracked from day 3 of exposure to the nitroimidazole inhibitors. Similarly, detailed analysis of the swim pattern of the larvae was performed at the end of day 5 after exposure to the inhibitors. For the analysis of movement pattern, the zebrafish larvae were placed in 35 × 15 mm Petri dish containing embryonic medium, and the larvae were allowed to settle in the Petri dish for 1 min. The movement of the zebrafish larvae was observed under the microscope for 1 min. The images of the moving zebrafish larvae were taken using a stereomicroscope, and the swim patterns were compared with the control group zebrafish larvae that were not treated with any inhibitor and the larvae treated with 1% DMSO.

#### Histochemical analysis

At the end of five days of inhibitor treatment, representative larvae from each group were collected for histological examination. The histological analyses were done to check the effect of inhibitors on internal tissues of the larvae that were treated with different concentrations of nitroimidazole compounds, and the results were compared to the control group larvae. At the end of the experiments the zebrafish larvae were washed with phosphate-buffered saline (PBS) and excess amounts of Tricaine were added to the wells to anesthetize the larvae. The anesthetized larvae were transferred to a 1.5 ml tube and fixed in buffered formaldehyde (formaldehyde solution 4%, pH 6.9) in PBS for 3 h at room temperature or overnight at 4 °C. After the fixation, the larvae were transferred to 70% ethanol and stored at 4 °C before being embedded in paraffin. The paraffin embedded samples were sectioned into 5 µm slices for histochemical staining. The fixed sections containing samples were deparaffinized in xylene, rehydrated in an alcohol series, and stained with Mayer’s Hematoxylin and Eosin Y (both from Sigma-Aldrich). After dehydration, the slides were mounted with Entellan^®^ Neu (Merck; Darmstadt, Germany). The slides containing the tissues were examined for the presence of pathological changes in the tissues of the larvae exposed to the inhibitors and photographed using a Nikon Microphot microscope (Nikon Microphot-FXA, Japan). All the procedures were carried out at room temperature unless stated otherwise.

#### Determination of minimal inhibitory concentration in in vitro cultures of Mycobacterium marinum

We determined the minimal inhibitory concentration (MIC) of the inhibitors according to the protocol by Hall et al.^33^ with some modifications. In short, wild type *M. marinum* (ATCC 927) was grown on Middlebrook 7H10 agar plates (BD) for six days at +29 °C. Bacterial mass was scraped from the plate and transferred into PBS pH 7.4 containing 0.2% Tween 80 (Sigma-Aldrich) to obtain an OD600 of 0.08–0.100. 200 ml of this bacterial suspension was mixed with 11 ml of Middlebrook 7H9 Broth OADC Becton Dickinson (BD) (no tween, no glycerol) by vortexing. The bacterial concentration was determined by plating on 7H10 agar (BD) and purity by plating on LB agar (Sigma-Aldrich). Plates were incubated for six days at +29 °C. The bacterial concentration was between 1.4 × 10^5^ and 4.7 × 10^5^ colony forming units (cfu)/ml. 50 µl of this bacterial suspension was pipetted per well onto sterile, clear 96-well tissue culture treated plates (Corning Costar from Sigma-Aldrich). The filter sterilized CA inhibitors dissolved in Middlebrook 7H9 Broth OADC (no tween, no glycerol) were added on top of bacteria in a volume of 50 µl. A concentration range of 200 µM–3 mM using a 10-fold dilution series was tested in two separate experiments on 6 replicate wells. The lids were sealed onto the plates with parafilm and the cultures were incubated at +28.5 °C for five days. The result was determined by assessing the turbidity of the cultures both by visual inspection and by an OD600 measurement using Perkin Elmer Envision multi-reader scan measurement. Five horizontal and five vertical points 0.72 mm apart were measured from each well. The sum of the readings was calculated for each sample. The background signal from wells containing medium only was subtracted from all values.

#### Constructs, oocytes and injections of cRNA

Human CA IX cDNA was kindly provided by Dr. Robert McKenna, University of Florida, Gainesville, and cloned into the oocyte expression vector pGEM-He-Juel, which contains the 5′ and the 3′ untranscribed regions of the *Xenopus* β-globin flanking the multiple cloning site. Plasmid DNA was transcribed *in vitro* with T7 RNA-Polymerase (mMessage mMachine, Ambion Inc., Austin, USA) as described earlier^27^^,^^34^. *Xenopus laevis* females were purchased from the Radboud University, Nijmegen, Netherlands. Segments of ovarian lobules were surgically removed under sterile conditions from frogs anesthetized with 1 g/l of ethyl 3-aminobenzoate methanesulfonate (MS-222, Sigma-Aldrich), and rendered hypothermic. The procedure was approved by the Niedersächsisches Landesamt für Verbraucherschutz und Lebensmittelsicherheit, Oldenburg, Germany (33.19-42502-05-17A113). As described earlier^27^^,^^34^, oocytes were singularized by collagenase (Collagenase A, Roche, Mannheim, Germany) treatment in Ca^2+^-free oocyte saline (pH 7.8) at 28 °C for 2 h. The singularized oocytes were left overnight in an incubator at 18 °C in Ca^2+^-containing oocyte saline (pH 7.8) to recover. Oocytes of the stages V and VI were injected with 5 ng of cRNA coding for human CA IX. The oocyte saline had the following composition: 82.5 mM NaCl, 2.5 mM KCl, 1 mM CaCl_2_, 1 mM MgCl_2_, 1 mM Na_2_HPO_4_, 5 mM HEPES; titrated with NaOH to the desired pH.

#### Determination of CA IX activity in Xenopus oocytes

Catalytic activity of CA IX in *Xenopus* oocytes was determined by monitoring the ^18^O depletion of doubly labelled 13C^18^O_2_ through several hydration and dehydration steps of CO_2_ and HCO_3_^−^ at 23 °C as previously described^35^^,^^36^. The reaction sequence of ^18^O loss from 13C^18^O^18^O (*m/z* = 49) over the intermediate product 13C^18^O^16^O (*m/z* = 47) and the end product 13C^16^O^16^O (*m/z* = 45) was monitored with a quadrupole mass spectrometer (OmniStar GSD 320; Pfeiffer Vacuum, Asslar, Germany). The relative ^18^O enrichment was calculated from the measured 45, 47, and 49 abundances as a function of time according to: log enrichment = log [49 × 100/(49 + 47 + 45)]. For calculation of CA IX activity, the rate of ^18^O degradation was obtained from the linear slope of the log enrichment over the time, using the spreadsheet analysing software OriginPro 8.6 (OriginLab Corporation, Northampton, USA). The rate was compared with the corresponding rate of the non-catalysed reaction. Enzyme activity in units was calculated from these two values as defined by^37^. From this definition, one unit corresponds to 100% stimulation of the non-catalysed ^18^O depletion of doubly labelled 13C^18^O_2_. The measurements were carried out in oocyte saline at pH 7.0. For every measurement the non-catalysed reaction was determined for 6 min before adding lysate from 20 CA IX-expressing oocytes to the cuvette. After the catalysed reaction was measured for 6 min, inhibitor was added stepwise in increasing concentration every 4 min. Catalytic activity in the presence of inhibitor was normalized to the catalyzed reaction in the absence of inhibitor for every measurement. IC_50_ values were calculated from a Hill1 fit using OriginPro 8.6.

#### Statistical analysis

The GraphPad Prism software (5.02) was used to perform statistical analysis. For statistical analysis of the toxicity parameters, a two-tailed Fisher’s test was used and *p* values below 0.05 were considered significant.

## Results

### Determination of LD_50_ values of DTP338 and DTP348

**Figure 2.**
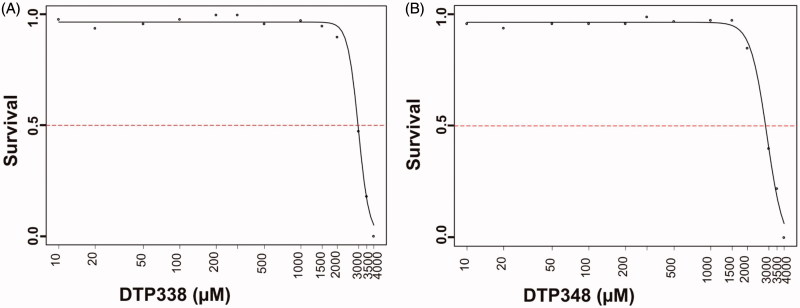
The LD_50_ determination of nitroimidazole inhibitors. The LD_50_ dose for the CA IX inhibitors DTP338 and DTP348 was determined based on cumulative mortality of the zebrafish larvae at the end of five days after the exposure of embryos to different concentration of the inhibitors. The LD_50_ doses for both the DTP338 and DTP348 compounds were in the range of 3.5 mM concentration (A and B). The LD_50_ were determined after three independent experiments with similar experimental conditions (*n* 180).

The toxic effects of both DTP338 and DTP348 compounds were dose-dependent in the zebrafish embryos that were exposed to either inhibitor for five days. Neither inhibitor showed any significant mortality up to 1.5 mM concentration at the end of five days of exposure to the inhibitors. The mortality of zebrafish embryos was dose-dependent the concentration required for the mortality of 50% of larvae at the end of five days of expose to the inhibitors was 3.5 mM for both inhibitors. The LD_50_ values of both inhibitors are shown in Figure 2.

### Phenotypic defects in the zebrafish larvae

**Figure 3.**
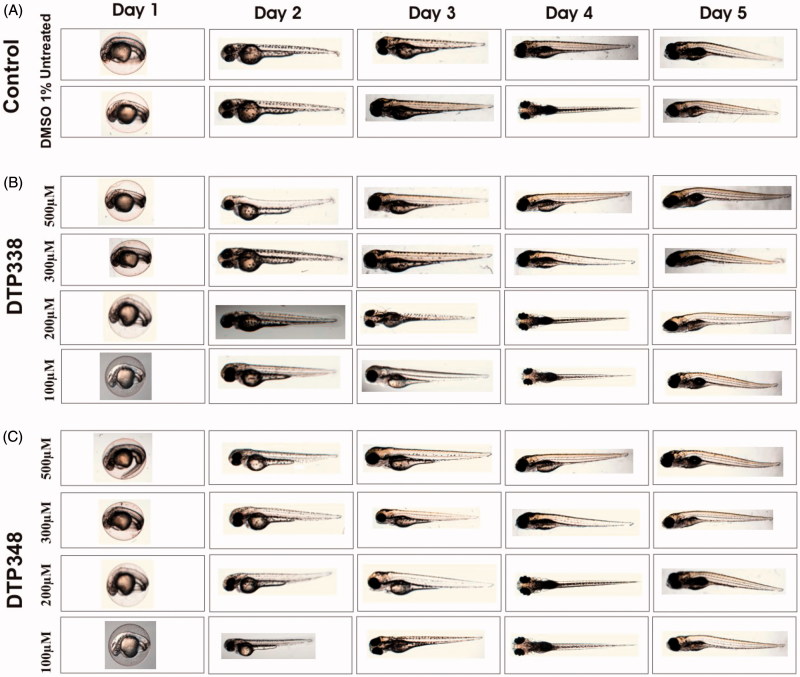
Phenotypic images zebrafish embryos in control and nitroimidazole inhibitor treated groups. Developmental images of 1–5 dpf zebrafish embryos exposed to different concentrations of DTP338 and DTP348. (A) The images of control group (not treated with inhibitors) and 1% DMSO treated zebrafish larvae show normal development. (B) The zebrafish larvae treated with different concentrations (100–500 μM) of DTP338. (C) The images of zebrafish larvae exposed to different concentrations (100–500 μM) of DTP348. The embryos exposed up to 500 μM concentrations show no apparent phenotypic abnormalities at the end of 5 after exposure to the drugs and had normal embryonic development.

To assess the adverse effects of the nitroimidazole derivatives on the developing zebrafish larvae we studied the observable phenotypic parameters of the larvae that were exposed to different concentrations of the inhibitors and compared with the zebrafish larvae that were exposed to 1% DMSO. Figure 3 shows images of representative larvae treated with 100–500 µM concentrations of the inhibitors and the images of larvae from control groups.

**Figure 4.**
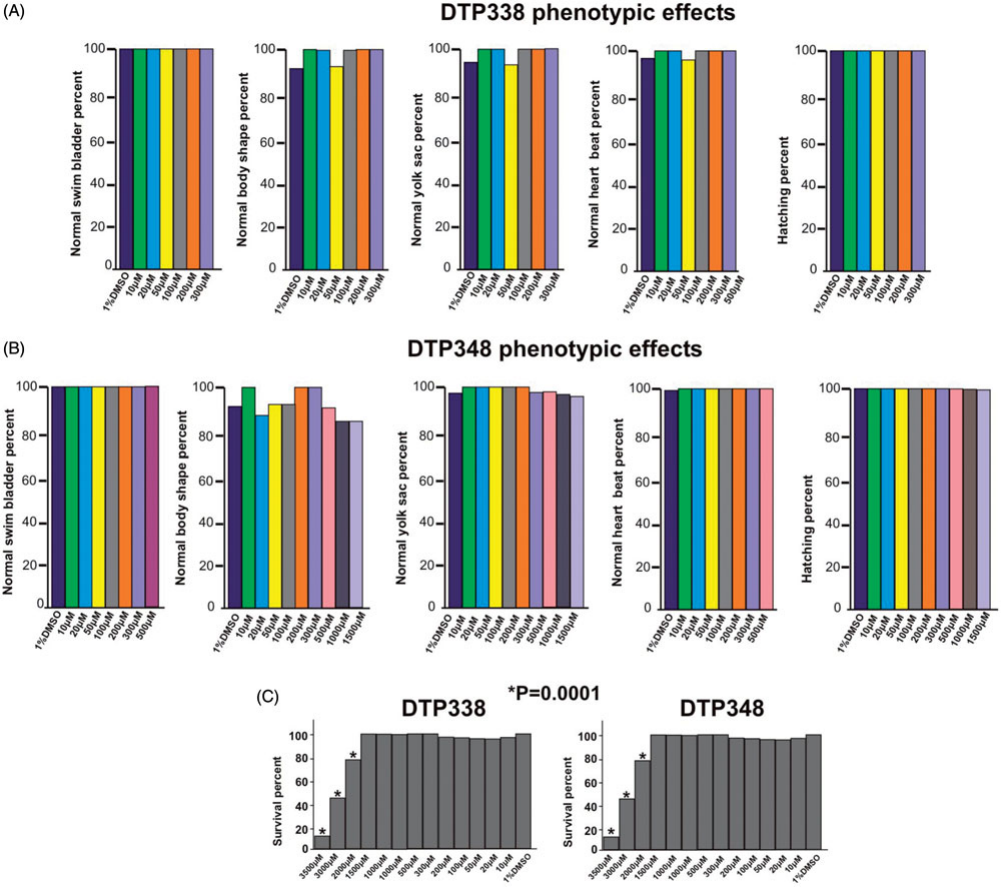
Effect of nitroimidazole inhibitors on phenotypic parameters of the 5 dpf zebrafish embryos. Panel A and B show bar graphs of observable phenotypic parameters (1) swim bladder, (2) body shape, (3) yolk sack, (4) heartbeat and (5) hatching and zebrafish larvae treated with different concentrations of DTP338 (A) and DTP348 (B). In the inhibitor treated groups of both the compounds, the parameters, swim bladder development, body shape, utilization of yolk sack, heartbeat and hatching showed no significant difference compared control group zebrafish larvae. At the end of five days after exposure to inhibitors the zebrafish larvae showed a significant difference in the swim pattern compared to control group larvae. (C) The bar graph panel shows the percent survival of the zebrafish larvae in inhibitor treated group and control group treated with 1% DMSO. For each concentration, n 120. *p < 0.05 by two-tailed Fisher’s test.

Neither inhibitors showed any significant adverse effects on the observable parameters, such as survival, hatching, oedema, heartbeat, body shape, swim bladder development, and yolk sac utilization at the concentration of 1.5 mM or below during the embryonic development, nor did they show any abnormalities at the end of five days of exposure compared to the embryos treated with 1% DMSO. Figure 4(A–C) shows the effects of inhibitors on the studied parameters and dose dependent mortality of the larvae.

### The zebrafish treated with inhibitors for five days showed ataxic movement pattern

**Figure 5.**
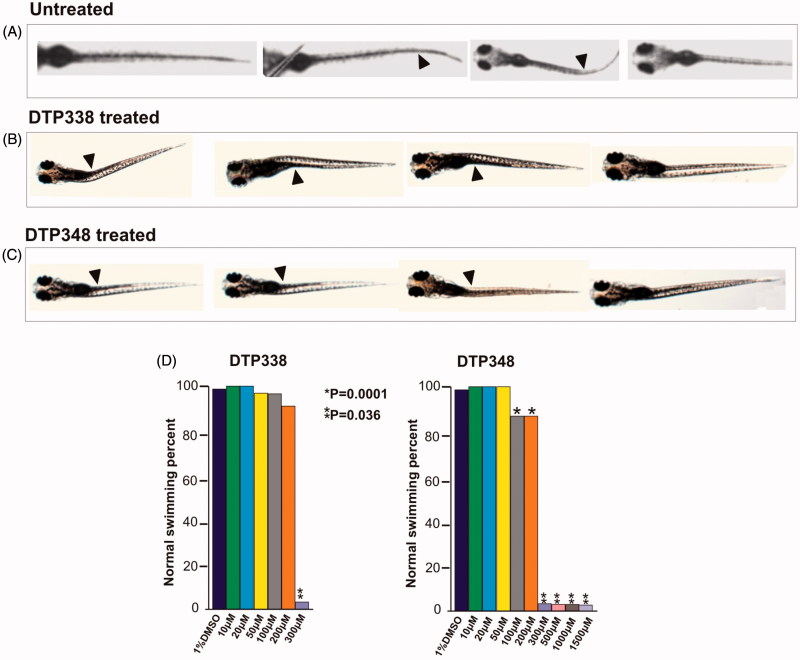
Ataxic swim pattern of the zebrafish larvae. (A) Zebrafish larvae (5 dpf) showing normal swim pattern. (B) The zebrafish larvae treated with 300 μM DTP338 show wobbling movement along the path at the end of five days after exposure to the inhibitor. (C) The zebrafish larvae treated with 300 μM DTP348 also show wobbling movement along the path at the end of fier days after exposure to the inhibitor. The swim pattern of the larvae treated with inhibitors showed difficulty in balancing the body while moving forward with a curve in the body (arrow heads). (D) Shows the bar graph of swim pattern treated with different concentration of nitroimidazoles.

The swim pattern of the zebrafish larvae was one of the parameters analysed for determination of toxic effects of the inhibitors. The analysis of the swim pattern showed no abnormality until 4 dpf. However, at the end of five days the larvae exposed to either DTP338 or DTP348 showed various degrees of ataxic movement patterns at the concentrations ranging from 100 to 1500 µM (Figure 5(D)). Further analysis of the swim pattern of the zebrafish larvae treated with 10–500 µM concentrations of the inhibitors showed significantly abnormal movement in the larvae treated with 300 µM or above concentration of DTP338 (*p* > 0.001). The lowest dose of DTP338 at which the zebrafish larvae showed some tendency of abnormal swimming was 200 µM. This difference of the swim pattern was not statistically significant compared to the control group embryos (Figure 5(D)). Interestingly, the zebrafish larvae treated with DTP348 showed considerably abnormal swim pattern even at 100 and 200 µM concentrations (*p* > 0.036) as shown in Figure 5(D). When compared with the control group larvae, the inhibitor treated larvae moved slowly and had increased turning angle with a difficulty in balancing the body when moving forward (Figure 5(A–C)).

### The histochemical analyses showed no effect on tissue morphology

**Figure 6.**
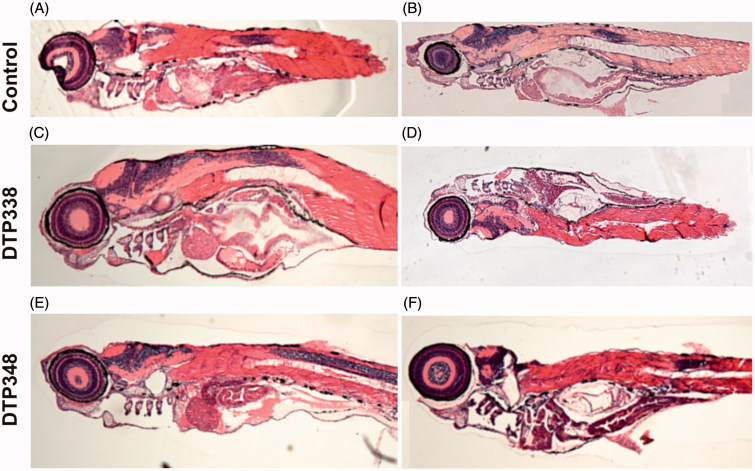
Histochemical images of control group and nitroimidazole inhibitors treated zebrafish larvae. (A) The image of larvae treated with 1% DMSO. (B) The image of zebrafish not treated with any drug. (C) The larvae treated with 2 mM concentration of DTP338. (D) The larvae treated with 500 μM concentration of DTP338. (E) The larvae treated with 2 mM concentration of DTP348. (F) The larvae treated with 500 μM concentration DTP348. The images presented here are selected from three independent groups of experiments. The sagittal sections show no apparent morphological changes or abnormalities in the tissues in the zebrafish larvae at the end of five days of exposure.

We studied the effect of these inhibitors on tissues by analysing thin sections of the zebrafish larvae stained with Eosin and Hematoxylin (H & E). The H & E stained sections of the inhibitor-treated zebrafish larvae were compared with the control group fish that were treated with 1% DMSO or not treated with any inhibitor. The microscopic examination of the sections did not reveal any damage to the tissues of the developing zebrafish larvae at the analysed concentrations of nitroimidazole inhibitors. The representative images of the fish that were analysed histochemically are shown in Figure 6.

### Determination of the minimal inhibitory concentration of nitroimidazole inhibitors

A study has shown that Ethoxzolamide, a general inhibitor of human CAs inhibits the PhoPR regulon, a two-component regulatory system in *Mycobacterium tuberculosis*, and prevents the formation of biofilms in Nontuberculous *mycobacteria* (NTM)^25^^,^^38^.

Therefore, we wanted to test if the nitroimidazole compounds DTP338 or DTP348 show any antibacterial effects in a zebrafish model. For this purpose, we utilized a well-characterized infection model where the fish are infected with *M. marinum*, a natural TB causing pathogen of zebrafish. First, we performed standard MIC experiments for both nitroimidazoles using liquid cultures of *M. marinum* on 96 well plates. In the MIC tests, we inspected growth of bacteria visually at the end of six days of incubation with different concentrations of the inhibitors. In addition, we measured the optical density of the cultures to obtain the growth curve of the bacteria. The experiments carried out twice with these inhibitors did not show any effects on the growth of *M. marinum* even at the concentrations as high as 2.5 mM. The MIC tests suggested that the nitroimidazole inhibitors do not inhibit the growth of bacteria and hence these inhibitors are not antibacterial (data not shown).

### *In vivo* inhibition of human CA IX activity in Xenopus oocytes

**Figure 7.**
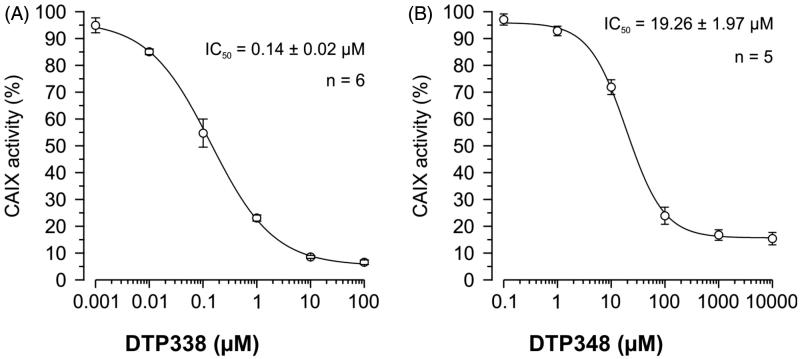
The IC_50_ doses of the two nitroimidazole compounds. (A) Shows the half maximal inhibition of human CA IX catalytic activity *in vivo* in *Xenopus* oocytes exposed to different concentrations of nitroimidazole DTP338. (B) Shows the half maximal inhibition of human CA IX catalytic activity *in vivo* in *Xenopus* oocytes exposed to different concentrations of nitroimidazole DTP348. The IC_50_ values were determined from five to six independent experiments.

The nitroimidazole inhibitors DTP338 and DTP348 inhibit the activity of recombinant human CA IX *in vitro* in nanomolar concentrations (Ki of 20.4 nM for DTP338 and Ki of 8.3 nM for DTP348) and are considered potential drugs for cancer therapy^18^. In addition to safety studies using zebrafish as a vertebrate model, we also aimed to determine the efficiency of these inhibitors for *in vivo* inhibition of CA activity. We used *Xenopus* oocytes as an *in vivo* model organism to determine the IC_50_ doses of the nitroimidazole compounds. The inhibition of the heterologously expressed human CA IX by DTP338 and DTP348 was monitored with mass spectrometry (MS). Among these two CA IX inhibitors, DTP338 inhibited the enzyme activity more effectively in the oocytes and the IC_50_ value for inhibition of CA IX was 140 nM (Figure 7(A)). The IC_50_ value of DTP348 (IC_50_ 19 µM) was higher compared to DTP338 (Figure 7(B)). Therefore, the nitroimidazole inhibitor DTP338 is potentially a better inhibitor of human CA IX compared to DTP348, at least *in vivo* in *Xenopus* oocyte model system.

## Discussion

The purpose of the study was to evaluate the safety and toxicity of novel nitroimidazole derivatives DTP338 and DTP348, and subsequently determine the *in vivo* inhibitory properties of these inhibitors against human CA IX in *Xenopus* oocytes. This is important in development of these inhibitors both as anticancer agents for the treatment of hypoxic tumours and as tumour sensitizers before treatment with existing treatment methods. Nitroimidazole inhibitors DTP338 and DTP348 belong to a structurally distinct class of CA inhibitors that have been designed and synthesized recently^18^. These compounds inhibit extracellular tumour acidification at least in two cell lines overexpressing CA IX, namely the colorectal HT-29 and the cervical HeLa carcinoma cell lines^18^. *In vitro* and *in vivo* studies have demonstrated that these inhibitors are potential drug candidates against human CA IX in the treatment of hypoxic tumours^18^^,^^39^. The lead compound of our study DTP348, that was used for sensitizing tumours in combination with doxorubicin in mice, was previously assessed for toxicity based only on the assessment of body weight measurements^18^^,^^39^. Interestingly, studies in the past have shown that the nitroimidazole compounds used for sensitizing tumours are highly toxic and unacceptable for use in therapy, even though they achieved therapeutic efficacy^21^^,^^22^.

Potential anti-cancer drugs need to be evaluated thoroughly for toxicity and safety prior to any tests in humans. To evaluate toxicity and doses required for inhibition of CA IX *in vivo*, we used both zebrafish larvae and *Xenopus* oocyte models, respectively. Zebrafish have been widely used for acute and chronic toxicity testing as well as for studying developmental toxicity^40^. Zebrafish embryos develop *ex utero* and the chemical compounds can be easily added to the fish tank water, making it a highly feasible model for studying many aspects of toxicity. Developing embryos are more easily affected by chemical compounds than adult fish making assays with embryos more sensitive^40^^,^^41^. *Xenopus* oocytes have been used for *in vivo* inhibition studies of CAs and for the determination of IC_50_ doses for the inhibition of human CA IX^42^.

For the evaluation of toxicity of the nitroimidazoles we used 1–5 dpf zebrafish and studied the effects of these inhibitors on mortality, hatching rate, oedema, heartbeat, swim bladder development, yolk sack and movement pattern. In addition, we studied histopathology of the tissues to investigate the toxic effect of the compounds on the tissues during embryonic development.

The toxicity screening of the inhibitors showed that the LD_50_ doses of both DTP338 and DTP348 were in the range of 3.5 mM. The studies also showed that the inhibitors did not exhibit any significant effects on the observable parameters namely hatching, yolk sac utilization, oedema, heartbeat, swim bladder development, and mortality when assessed at 1.5 mM concentration after five days of exposure to the inhibitors. Histochemical staining of the whole zebrafish sections showed no apparent damage to the tissues in the larvae exposed to inhibitor concentrations below 2 mM (Figure 6(C–F)).

Interestingly, embryos exposed to either of the inhibitors at a concentration of 100 µM or above generally showed mild to significant ataxic swim pattern (abnormal movement), which is likely caused by neurotoxicity, at the end of five days exposure. This is similar to the swim pattern of fish which have the *CA8* gene knocked down, a gene associated with motor coordination function in human, mice and zebrafish^43^. Apart from ataxic swim pattern none of the fish showed significant defects in morphologic phenotype. However, further studies using molecular and electron microscopic techniques could reveal the precise changes in the brain. Based on the results of the present study, it is important to consider the dose of the inhibitors critical either for sensitizing the tumours or for the treatment of the solid tumours.

The IC_50_ value of DTP338 for human CA IX expressed in *Xenopus* oocytes was 140 nM, while it was 19.26 µM in the case of DTP348. The current *in vivo* inhibition results contrast with the results of *in vitro* inhibition study reported earlier^18^. These current results suggest that the requirement of therapeutic doses for the inhibition of CA IX *in vivo* in tumours may be higher than suggested by *in vitro* studies, and need to be carefully considered when deciding the therapeutic doses for the treatment of cancers. The obtained values will also help to define more precisely the concentrations for further pre-clinical testing in rodent models.

The nitroimidazole compounds have been designed and synthesized for inhibiting human CA IX and *in vitro* inhibition analysis using human recombinant CA IX previously showed that these inhibitors inhibit hCA IX in sub-nanomolar quantities^18^. We hypothesized that, if these inhibitors are not toxic and inhibit only human CAs, they should not inhibit the growth of the *M. marinum* bacteria, neither due to toxicity nor via inhibition of β-CAs critical for their survival. In our earlier study, the growth of the mycobacterium could be inhibited *in vitro* in culture and *in vivo* in zebrafish using dithiocarbamates, the known inhibitors of mycobacterial β-CAs^25^. The MIC tests of the current study showed that these nitroimidazole compounds do not inhibit the growth of the bacterium even at concentrations as high as 2.5 mM, suggesting that these inhibitors are not toxic to the bacterium, neither directly nor through the inhibition of β-CAs.

In conclusion, this study showed that the nitroimidazole inhibitors DTP338 and DTP348 exhibit no toxicity at or below 300 µM concentrations during the embryonic development of zebrafish, and hence are probably safe at lower concentrations for use as anticancer drugs. Although these inhibitors inhibit the activity of human CA IX *in vitro* at low nanomolar concentrations, the concentration required for the inhibition of human CA IX *in vivo* is low micromolar. The observed discrepancy between the *in vitro* and *in vivo* results is interesting, indeed, and there might be several potential explanations for this difference. Other proteins may interfere with inhibitor binding *in vivo* and thus change the pharmacokinetic properties of the inhibitors. On the other hand, the chemical stability of the inhibitor may be affected *in vivo* due to the presence of multiple biological and chemical factors. Nevertheless, our result suggests that the required concentration for inhibition of CA IX in human cells may be at micromolar range. Because the zebrafish studies suggested some neurotoxicity at higher concentration levels, further preclinical *in vivo* studies are warranted to adjust optimal inhibitor concentrations before entering any clinical trial.
